# Evaluation of 2'-Deoxy-2'-fluoro Antisense Oligonucleotides for Exon Skipping in Duchenne Muscular Dystrophy

**DOI:** 10.1038/mtna.2015.39

**Published:** 2015-12-01

**Authors:** Silvana M G Jirka, Christa L Tanganyika-de Winter, Joke W Boertje-van der Meulen, Maaike van Putten, Monika Hiller, Rick Vermue, Peter C de Visser, Annemieke Aartsma-Rus

**Affiliations:** 1 Department of Human Genetics, Leiden University Medical Center, Leiden, The Netherlands; 2 BioMarin, Leiden, The Netherlands

**Keywords:** antisense oligonucleotide, 2'-deoxy-2'-fluoro, exon skipping, 2'-O-methyl phosphorothioate, dystrophin

## Abstract

Duchenne muscular dystrophy (DMD) is a severe muscle wasting disorder typically caused by frame-shifting mutations in the *DMD* gene. Restoration of the reading frame would allow the production of a shorter but partly functional dystrophin protein as seen in Becker muscular dystrophy patients. This can be achieved with antisense oligonucleotides (AONs) that induce skipping of specific exons during pre-mRNA splicing. Different chemical modifications have been developed to improve AON properties. The 2'-deoxy-2'-fluoro (2F) RNA modification is attractive for exon skipping due to its ability to recruit ILF2/3 proteins to the 2F/pre-mRNA duplex, which resulted in enhanced exon skipping in spinal muscular atrophy models. In this study, we examined the effect of two different 2'-substituted AONs (2'-F phosphorothioate (2FPS) and 2'-*O*-Me phosphorothioate (2OMePS)) on exon skipping in DMD cell and animal models. In human cell cultures, 2FPS AONs showed higher exon skipping levels than their isosequential 2OMePS counterparts. Interestingly, in the *mdx* mouse model, 2FPS was less efficient than 2OMePS and suggested safety issues as evidenced by increased spleen size and weight loss. Our results do not support a clinical application for 2FPS AON.

## Introduction

Duchenne muscular dystrophy (DMD) is a severe X-linked muscle-wasting disorder affecting 1 in 5,000 newborn boys.^1,2^ DMD is caused by out-of-frame or nonsense mutations in the *DMD* gene that lead to a truncated, nonfunctional dystrophin protein. Dystrophin is an important shock-absorbing protein in muscle and without it, muscles are easily damaged. Restoration of the reading frame in DMD patients would in theory allow the production of a shorter, but partly functional dystrophin protein as seen in less severely affected Becker muscular dystrophy patients.^3,4^ This can be achieved with antisense oligonucleotides (AONs) that target and induce skipping of specific exons during pre-mRNA splicing.^5,6^ Exon skipping AONs are thought to act by sterically hindering splicing factors in the recognition of the exon and/or splicing sites.

Over the years, chemical modifications have been developed to improve AON characteristics, such as improved binding affinity to the target transcript, increased resistance against nuclease degradation and improved cellular uptake.^7^ Two different AON chemistries, phosphorodiamidate morpholino oligonucleotides and 2'-*O*-methyl phosphorothioate (2OMePS), are currently in clinical development for exon skipping in DMD.^8,9,10,11,12^

The 2'-deoxy-2'-fluoro (2F) chemistry may also be an attractive chemistry for exon skipping AONs. Recently, it has been shown that the duplex of 2F AON and its target pre-mRNA attracts interleukin enhancer binding factors 2 and 3 (ILF2/3 proteins) resulting in unanticipated exon skipping in a model of spinal muscular atrophy.^13^ Probably this is based on enhanced steric hindrance by the duplex/protein complex, which impedes binding of splicing factors to splice sites or exonic regions in the pre-mRNA transcript beyond the AON target sequence. As enhanced exon skipping is relevant for DMD therapeutics, we compared the efficiencies of isosequential 2'-F phosphorothioate (2FPS) and 2OMePS AONs targeting exonic regions within different human dystrophin exons, or in the 5' donor splice site of mouse dystrophin exon 23 (**Supplementary Figure S1**). In *in vitro* transfection experiments, 2FPS AONs outperformed their 2OMePS counterparts, while *in vivo* they appeared less effective.

## Results

### *In vitro* evaluation

**Figure 1:**
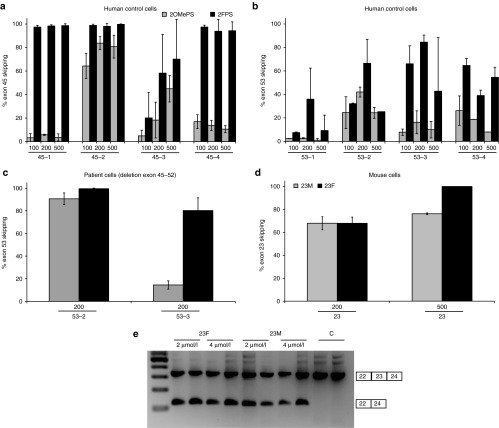
**Exon skip evaluation of human and mouse myotube cultures**. RT-PCR analysis of human and mouse myotubes transfected with 100–500 nmol/l of 2OMePS or isosequential 2FPS AON (*n* = 4). (**a**) Exon 45 AONs in control myotubes. (**b**) Exon 53 AONs in control myotubes. (**c**) Exon 53 AONs in Duchenne muscular dystrophy patient-derived (Δ45–52) myotubes. (**d**) Mouse exon 23 AONs in mouse myotubes. (**e**) Exon 23 skipping in primary mouse myoblasts without the use of a transfection reagent (gymnotic delivery). Bars represent means ± SD.

To test whether 2FPS AONs are capable of inducing dystrophin exon skipping, human control myotube cultures were transfected with 100–500 nmol/l of several 2FPS AONs and their isosequential 2OMePS counterparts.^14^ These AONs have different activity profiles and target exon 45 or exon 53 (**Supplementary Table S1**, **Figure 1**). RNA was isolated after 48 hours and exon-skipping levels were determined semiquantitatively by lab-on-a-chip analysis after nested reverse transcription- PCR (RT-PCR) amplification. We observed highest exon 45 skipping levels for each of the 2FPS AONs, with three out of four of the 2FPS AONs having exon skipping levels over 90% at all concentrations tested (**Figure 1a**). For exon 53 skipping, although percentages were more variable, all 2FPS AONS induced relatively higher exon-skipping levels than their 2OMePS AON counterparts (**Figure 1b**). This effect was also confirmed in DMD patient-derived Δ45–52 myotube cultures, in which skipping of exon 53 is frame-restoring and potentially therapeutic (**Figure 1c**).

We also evaluated the potential of 2FPS AONs targeting mouse dystrophin exon 23 in mouse control myotube cultures.^15^ Upon the use of a transfection reagent, we observed a slight increase in exon 23 skipping with 2FPS AON (23F) compared to the 2OMePS AON (23M) at 500 nmol/l. However, no differences between 23F and 23M were observed at 200 nmol/l (**Figure 1d**). Finally, we also tested the activity of 2FPS AON in primary myoblasts derived from e*xtensor digitorum longus* muscles of an *mdx* mouse, a mouse model for DMD.^16^ In this case, we did not use a transfection reagent (“gymnotic delivery”). Primary myoblasts were incubated with 2 or 4 µmol/l of 23M or 23F AON at initiation of differentiation into myotubes. After 96 hours RNA was isolated and analyzed by nested RT-PCR. Exon 23 skipping was confirmed for both AONs at comparable levels (**Figure 1e**).

### *In vivo* evaluation

**Figure 2:**
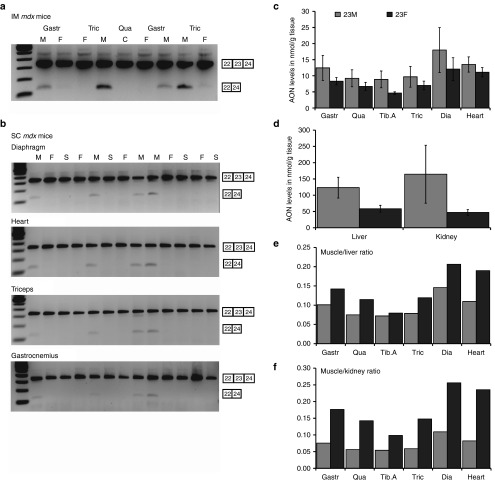
**Exon skipping and pharmacokinetic analysis of 23F- and 23M-treated *mdx* mice**. (**a**) RT-PCR analysis of muscles from two *mdx* mice, intramuscularly injected with 2.9 nmol of 23M or 23F contralaterally for 2 consecutive days. (**b**) RT-PCR analysis of skeletal and heart muscles isolated from *mdx* mice (four to five mice/group) subcutaneously treated four times per week with 50 mg/kg of 23M, an equimolar amount of 23F or saline for 8 weeks. (**c**) Antisense oligonucleotide (AON) concentrations in skeletal muscles and heart assessed with a hybridization ligation assay. (**d**) AON concentrations in liver and kidney as assessed with a hybridization ligation assay. (**e**) Ratios of AON levels in muscle compared to kidney. (**f**) Ratios of AON levels in muscle compared to liver. C, untreated control; Dia, *diaphragm*; F, 23F; Gastr, *gastrocnemius*; M, 23M; S, saline; Qua, *quadriceps*; Tib.A, *tibialis anterior*; Tric, *triceps* (*T-test for significant *P* < 0.05). Bars represent means ± SD.

Two *mdx* mice were intramuscularly (IM) injected with 2.9 nmol of 23M or 23F AON for 2 consecutive days in *gastrocnemius* and *triceps* muscles. One week after the last injection the mice were sacrificed and the injected muscles harvested for RNA isolation. Exon skipping was determined by nested RT-PCR and visualized on an agarose gel. Surprisingly no clear exon skipping could be detected for the 23F AON, in contrast to the 23M AON (**Figure 2a**).

In parallel, we evaluated subcutaneous administration of the 2FPS AON targeting mouse exon 23. Groups of 4–5 *mdx* mice were administered 23M, 23F AON or saline, through 4 weekly injections of 50 mg/kg for 23M AON, or molar equivalent for 23F AON for 8 weeks. One week after the last injection the mice were sacrificed and various muscles and other tissues were harvested for analysis. Exon skipping was assessed for skeletal- and cardiac muscles by single round RT-PCR and visualized on an agarose gel. Surprisingly, again no exon skipping could be detected in 23F AON-treated mice in any of the muscles analyzed, while exon skipping was detectable in the case of 23M AON-treated mice for each muscle analyzed (**Figure 2b**). As anticipated, dystrophin restoration was observed by western blot for 23M AON-treated mice, but not for 23F AON or saline-treated mice (**Supplementary Figure S2**).

Assessment of the AON concentrations in different organs revealed lower levels of 23F AON in skeletal and cardiac muscle, liver, and kidney compared to 23M AON-treated animals (**Figure 2c**,**d**). The calculated target tissue muscle/kidney and muscle/liver ratios were higher for 23F AON suggesting that while uptake in muscle is lower for 23F than 23M AON, it is even further reduced in kidney and liver (**Figure 2e**,**f**).

**Figure 3:**
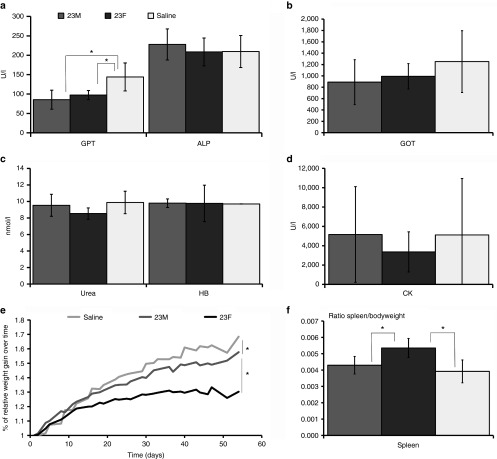
**Analysis of plasma protein levels and weight of 23F- and 23M-treated *mdx* mice**. (**a**) Glutamate pyruvate transaminase, alkaline phosphates, (**b**) glutamic oxaloacetic pyruvate transaminase, (**c**) hemoglobin, urea, (**d**) creatine kinase, and (**e**) weight gain over time; (**f**) ratio spleen/bodyweight. Bars represent means ± SD

Blood and plasma were determined for markers of liver and kidney damage and function as part of the safety profiling of the AONs. Glutamic oxaloacetic pyruvate transaminase and glutamate pyruvic transaminase are both enzymes that leak into the bloodstream upon liver and muscle damage. No significant differences were observed for glutamic oxaloacetic pyruvate transaminase, while significantly lower glutamate pyruvic transaminase levels were found for mice treated with either AON compared to saline-treated mice. Alkaline phosphatase (a marker for hepatobiliary function), urea (a marker for kidney function), and hemoglobin levels showed no significant differences between the three groups and were in the normal range for *mdx* mice. Large variations in individual levels of creatine kinase, an enzyme that leaks into the bloodstream upon muscle damage, prevented comparisons between the groups (**Figure 3a**–**d**).

Notably, 23F AON-treated mice had significantly lower increases in body weight over time compared to 23M AON and saline-treated mice (**Figure 3e**). Additionally, we found a significantly higher spleen/bodyweight ratio for mice treated with 23F AON compared to the other groups at sacrifice (**Figure 3f**).

**Figure 4:**
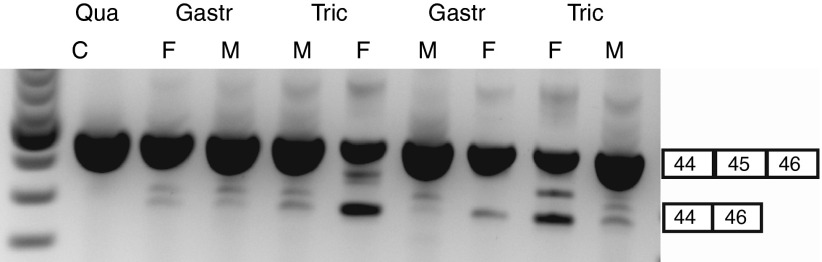
**Exon skip evaluation of intramuscular injection of 2FPS antisense oligonucleotides (AON) and 2OMePS counterparts in hDMD mice**. Two hDMD mice were intramuscularly injected with cardiotoxin 2 days prior to AON treatment of 2.9 nmol with 2OMePS or 2FPS AON counterpart targeting human exon 45 for 2 consecutive days. RNA was isolated 1 week after the last injection, subjected to RT-PCR and exon skipping visualized on an agarose gel. C, untreated control; F, 23F; Gastr, *gastrocnemius*; M, 23M; Qua, *quadriceps*.

To assess whether the discrepancy between *in vitro* and *in vivo* results observed for mouse exon 23 AONs also occurred for other exons, we compared a 2FPS and 2OMePS AON targeting human exon 45 *in vivo* in the hDMD mouse model. This mouse model carries the human *DMD* gene integrated in the mouse genome, which compensates for lack of mouse dystrophin resulting in healthy muscle.^17^ Since AON uptake in *mdx* mice is facilitated by the dystrophic phenotype, we pretreated hDMD *gastrocnemius* and *triceps* muscles with IM cardiotoxin injections to induce muscle necrosis and enhance AON uptake.^18^ Two days later treated muscles were injected with 2.9 nmol of the most potent 2OMePS AON targeting human exon 45 (45-2M) or isosequential 2FPS AON (45-2F) for 2 consecutive days into *gastrocnemius* and *triceps* muscles. One week after the last injection, RNA was isolated, and exon skip levels were determined by nested RT-PCR and visualized on an agarose gel. Results suggested enhanced exon skipping for 2FPS AON over the 2OMePS AON, which was most pronounced in the *triceps* muscle (**Figure 4**). These results demonstrate that 2FPS AONs are in fact capable of exon skipping *in vivo* after IM injections and the failure of the 2FPS AON to induce mouse dystrophin exon 23 skipping *in vivo* is not due to an inability of 2FPS AONs to be active *in vivo* per se.

## Discussion

Exon skipping is a therapeutic approach using AONs to reframe dystrophin transcripts for DMD and is currently evaluated in clinical trials.^11^ 2FPS AONs have shown unanticipated enhanced exon skipping in a model of spinal muscular atrophy due to recruitment of ILF2/3 proteins to the 2F/pre-mRNA duplex.^13^ For DMD, exon skipping is a desired feature, making 2FPS AONs potentially useful tools for reframing dystrophin transcripts. In this study, we demonstrate *in vitro* that 2FPS AONs have enhanced exon skipping in human and mouse myotube cultures over 2OMePS AON counterparts. The increased efficiency was most pronounced for human exon 45 AONs and least for mouse exon 23 AONs. A possible explanation *in vitro* is the difference in AON target sites and parameters such as AON sequence composition and secondary structure of the target region. For example, the AONs used here to target human exon 45 or 53, target intraexonic sites whereas the mouse AON targets a donor splice site. It is likely that the added bulkiness of recruited ILF2/3 by fluoro modified AONs is less effective when targeting exon intron boundaries where direct competition takes place with U1 snRNP binding sites than when targeting predicted ESE in intraexonic regions. This is consistent with our previous finding that exonic AONs appear to outperform splice site targeting AONs.^6^

*In vivo* we demonstrated that 2FPS AONs were not capable of skipping exon 23 in *mdx* mice, in contrast to 2OMePS AONs. A possible explanation for the observed difference between 2OMePS and 2FPS AON *in vivo* is that uptake of 2FPS by skeletal muscles after systemic treatment in *mdx* mice yielded insufficient concentrations to allow exon skipping. However, this does not explain why no exon 23 skipping was detected after IM injections in *mdx* mice. Potentially lower *in vivo* stability of 2FPS AONs could lead to lower tissue levels, but the fact that human exon 45 skipping 2FPS AONs were effective *in vivo* after IM injections argues against this possibility. It should be noted, however, that the exon 45 AON was tested in healthy muscle, while it is possible that in the *mdx* mouse the underlying pathology, such as the chronic inflammation and high muscle turnover caused increased 2FPS degradation.

Recently, Shen *et al*.^19^, showed *in vitro* that 2FPS modified AON interfered with splicing proteins and that 2FPS AON treatment of cultured cells resulted in a general disruption of normal splicing. We evaluated the accuracy of dystrophin splicing for exons 46 to 53 and the splicing of other genes (β-2-Microglobulin, transforming growth factor β1, α-1 type I collagen and Activin, chosen because of their involvement in immunogenicity, proliferation, differentiation, etc.) in muscle RNA isolated from saline, 2OMePS- and 2FPS-treated mice, but did not observe any differences between the groups (data not shown). This suggests that the splicing disruptions as observed by Shen *et al.*, might not underlie the lack of exon 23 skipping we observed in the *mdx* mouse. However, only a deep analysis of the full dystrophin transcript can rule out the occurrence of splicing abnormalities completely.

Since the first encouraging 2F results were published more than 20 years ago,^20^ only short-term *in vivo* experiments (1–3 weeks) have been reported for 2F-modified AONs. Furthermore, only a limited number of tissues were evaluated after intraperitoneal administration in spinal muscular atrophy^13^ or normal mice.^21^ To the best of our knowledge, no long-term *in vivo* follow-up studies have ever been reported for this chemistry.

With respect to safety, in our study of systemic AON treatment targeting mouse exon 23, no clear indications of toxicity were seen in markers for liver, kidney, or muscle damage. However, mice treated with 2FPS AON had a significantly higher spleen/bodyweight ratio compared to 2OMePS AON- and saline-treated mice. This corroborates the finding^21^ of a dose-dependent increase in spleen weights for mice treated with 2F AONs with flanking and or alternating 2'-*O*-methoxyethyl nucleotides after three weeks treatment with 6.25–50 mg/kg twice a week. Lastly, we also noticed that the 2FPS-treated *mdx* mice gained significantly less bodyweight than saline and 2OMePS-treated mice. On average, the 2FPS-treated mice weighed 17% less at the end of our experiment. Taken together, this suggests that *mdx* mice did not tolerate the treatment with fully modified 2FPS AONs targeting mouse exon 23 very well.

In summary, our data shows that 2FPS modified AONs had an improved effect *in vitro* and were effective *in vivo* on *DMD* exon skipping when targeting human exons, but this effect was minimal or absent *in vitro* and *in vivo* targeting mouse *DMD* exon 23. The exact reason for a lack of exon skipping *in vivo* by 2FPS modified AON targeting mouse *DMD* exon 23 is still unclear. In addition, 2FPS AONs revealed possible safety issues for long-term *in vivo* application, which needs to be further addressed when one wants to use such AONs in future studies. However, our results do not support a clinical application for 2FPS AON.

## Materials and Methods

*AONs.* Sequences of 2FPS and 2OMePS AONs are provided in **Supplementary Table S1**. 2FPS AONs contained 2'-deoxy-2'-fluoro RNA with a phosphorothioate backbone (ChemGenes corporation, Wilmington, MA). 2OMePS AONs consisted of 2'-*O*-methyl RNA with a phosphorothioate backbone (BioMarin, Leiden, the Netherlands).

*Cell culture*

*Human myoblasts.* Primary human control myoblasts and patient myoblasts with a deletion of exon 45–52 were a kind gift from Vincent Mouly.^22^ Cells were grown in skeletal muscle cell growth medium (Promocell, C-23160, Heidelberg, Germany) supplemented with an additional 15% fetal bovine serum (Gibco-BRL, Bleiswijk, the Netherlands) and 50 µg/ml gentamicin (PAA Laboratories, Germany/the Netherlands) in uncoated flasks until 70–80% confluence was reached. Cells were plated in a six-well plate coated with 0.5% gelatin (Sigma Aldrich Chemie B.V., Zwijndrecht, the Netherlands), at a density of 10^5^ cells per well, 48 hours prior to differentiation. Reaching 90% confluence, medium was switched to differentiation medium (Dulbecco's medium (without phenol red) with 2% fetal bovine serum, 50 μg/ml gentamicin, 2% glutamax, and 1% glucose (all from Gibco-BRL, Bleiswijk, the Netherlands)). Cells were allowed to differentiate for 4–5 days.

*Mouse myoblasts.* Mouse myoblasts were grown in Dulbecco's medium (without phenol red) supplemented with 10% fetal bovine serum, 1% Penicillin/Streptomycin (P/S), 2% Glutamax, and 1% glucose (all from Gibco-BRL) in collagen coated flasks. Cells were seeded in collagen coated six-well plates with proliferation medium and grown until confluence. The cells were washed twice with Hank's balanced salt solution and differentiation medium, Dulbecco's medium (without phenol red) supplemented with 2% horse serum, 1% P/S, 2% glutamax, and 1% glucose (all from Gibco-BRL) was added to induce differentiation. The cells were differentiated for 7–9 days before AON transfection.

*Primary myoblasts from mdx mice.* Primary myoblasts were isolated from the *extensor digitorum longus* muscle of one *mdx* mouse by collagenase treatment followed by single fiber isolation.^23^ Fibers were grown in SC+ medium (Dulbecco's medium (without phenol red) supplemented with 10% (horse serum), 30% fetal calf serum, 1% chicken embryonic extract (all from Gibco-BRL) and 10 μl/30 ml final medium of fibroblast growth factor (Promega Benelux B.V. Leiden, the Netherlands) on matrigel (GFR Matrigel BD Biosciences, San Jose, CA) coated plates. After 3 days, myoblasts were separated from fibroblasts by pre-plating in proliferation medium (Dulbecco's medium (without phenol red) supplemented with 10% fetal bovine serum, 1% P/S, 2% glutamax, and 1% glucose (all from Gibco-BRL). Finally, primary myoblast cells were plated in 12-well plates at 70–80% confluence; the following day, the cells were washed with phosphate-buffered saline (PBS) and differentiation medium (Dulbecco's medium (without phenol red) supplemented with 2% horse serum, 1% P/S, 2% glutamax, and 1% glucose (all from Gibc-BRL)) was added.

In vitro *delivery*

*AON transfection of myotube cultures.* Primary human control, patients myotube, and mouse myotube cells were transfected with either 100, 200, or 500 nmol/l of AONs using 6 µl of Lipofectamin 2000 (according to manufacturer's protocol) per well. After incubation for 3–4 hours at 37 °C and 5% CO_2_, cells were washed twice with PBS and 2 ml of fresh differentiation medium was added. Forty-eight hours later, RNA was isolated.

*Gymnotic delivery.* Primary myoblasts from *mdx* mice were incubated with 2 or 4 µmol/l of 23M or 23F AON for 96 hours at initiation of differentiation.

In vitro *delivery*

*Intramuscular injection of mdx mice.* Two *mdx* mice were IM injected in the *gastrocnemius* and *triceps* muscles with 2.9 nmol of 23M (20 µg) or 23F AON in contralateral muscles for 2 consecutive days. One week after the last injection the mice were sacrificed, and *quadriceps* (noninjected control), *gastrocnemius*, and *triceps* muscles were isolated.

*Intramuscular injection of hDMD mice.* Two hDMD mice were injected in the *gastrocnemius* and *triceps* muscles with cardiotoxin 2 days prior to injection with 2.9 nmol of 23AON or 2FPS23AON contralateral for 2 consecutive days. One week after the last injection the mice were sacrificed, *quadriceps* (noninjected control), *gastrocnemius*, and *triceps* muscles were isolated.

*Systemic treatment in mdx mice.* Five-week-old *mdx* mice (4–5 mice per group) were subcutaneously injected four times per week, with 50 mg/kg of 23M AON in 100 µl of saline or the molar equivalent (6.8 µmol) for 23F AON. *Mdx* mice were treated for 8 weeks and sacrificed 1 week after the last injection. All mice were weighed prior to injection and at the day of sacrifice. Blood samples were obtained from the tail vein for plasma pharmacokinetics analysis at the day of sacrifice. *Gastrocnemius*, *quadriceps*, *tibialis anterior*, *triceps* and *diaphragm* muscles, heart, liver, and kidney were isolated to determine exon skipping levels and AON concentrations. The spleen was isolated and weighed.

*RNA isolation*

*RNA isolation and cDNA synthesis of myotube cultures.* Human and mouse cells were washed twice with PBS. RNA was isolated by adding 500 µl TriPure (Roche diagnostics, Woerde, the Netherlands) to each well to lyse the cells. This was followed by chloroform extraction in a 1:5 ratio on ice for 5 minutes. The remaining cell debris was spin down by centrifugation (4 °C, 15 minutes, 15,4000 rcf) and the upper aqueous phase precipitated for 30 minutes on ice with equal volume of isopropanol. The RNA/isopropanol precipitate was centrifuged (4 °C, 15 minutes, 15,400 rcf) and the pellet was washed with 70% ethanol. The final RNA pellet was dissolved in 15 µl of RNase/DNase free water. For complementary DNA (cDNA) synthesis, 11 µl of RNA was used in a 20 µl reaction with a specific reverse primer (in human exon 48 for evaluating exon 45 skipping, in human exon 56 for evaluating exon 53 skipping, in mouse exon 26 for evaluating exon 23 skipping) and transcriptor reverse transcriptase (Roche Diagnostics) for 30 minutes at 55 °C and 5 minutes at 85 °C to terminate the reaction according the manufacturer's instructions.

*RNA isolation and cDNA synthesis of tissue.* Samples were homogenized in TriPure solution using a MagNA Lyser and MagNA Lyser green beads. Total RNA was isolated and purified according manufacturer's instructions. For the cDNA synthesis, 400 ng of RNA was used in a 20 µl reaction with random hexamers and transcriptor reverse transcriptase (all from Roche diagnostics, Woerde, the Netherlands) for 45 minutes at 42 °C and put on ice.

In vitro *exon skip evaluation.* Exon skipping was determined by nested RT-PCR. For RT-PCR analysis 3 µl of cDNA was incubated with 0.625 U AmpliTaq polymerase (Roche Diagnostics), 10 pM of primers (in exon 43 and 48 for exon 45 skipping, for exon 53 skipping in exon 56 and 43 (patient) or exon 50 (control), and mouse exon 21 and 26 for mouse exon 23 skipping) 5pmol of dNTPs and one time Supertaq PCR buffer (Sphaero-q, Gorinchem, the Netherlands) and amplified for 20 cycles each consisting of 40 seconds at 94 °C, 40 seconds at 60 °C, and 80 seconds at 72 °C. This PCR was followed by a nested PCR. For the nested PCR analysis 1.5 µl of the first PCR product was incubated with 1.25 U AmpliTaq polymerase (Roche Diagnostics), 20 pmol of primers (human exon 44 and 46 for evaluating exon 45 skipping, human exon 55 and 44 (patient) or 51 (control for evaluating exon 53 skipping, and mouse exon 22 and 24 for exon 23 skipping) 10 pmol of dNTPs and 1 times Supertaq PCR buffer and amplified for 32 cycles each consisting of 40 seconds at 94 °C, 40 seconds at 60 °C, and 60 seconds at 72 °C. PCR fragments were analyzed using 1.5% agarose gel electrophoresis. Exon skip levels were semiquantitatively determined as the percentages of the total (wild type and skipped) product with the Calipur LabChip GX (PerkinElmer, Groningen, the Netherlands).

In vitro *exon skip evaluation*

*Exon skip evaluation of intramuscular-injected mice.* Exon skipping was determined by nested RT-PCR and visualized on an agarose gel as described for the myotubes cultures detecting exon 45 skipping (hDMD) or exon 23 skipping (*mdx* mice).

*Exon skip evaluation of systemically treated mdx mice.* Exon skipping was determined by single RT-PCR. For RT-PCR analysis 1.5 µl of cDNA was incubated with 1.25 U taq polymerase (Roche Diagnostics), 20 pmol of primers (reverse primer in exon 24, forward primer in exon 22) 10 pmol of dNTPs and one time Supertaq PCR buffer (Sphaero-q) and amplified for 30 cycles each consisting of 30 seconds at 94 °C, 30 seconds at 60 °C, and 30 seconds at 72 °C. PCR fragments were analyzed by 2% agarose gel electrophoresis.

In vitro *safety and quantification of AON levels*

*Plasma parameters.* Blood was collected in lithium-heparin-coated microvettes CB300 (Sarstedt B.V., Etten-Leur, the Netherlands). Glutamate pyruvate transaminase, alkaline phosphates, glutamic oxaloacetic pyruvate transaminase hemoglobin, urea, and creatine kinease were determined using Reflotron strips in the Reflotron Plus machine (Roche Diagnostics).

*Quantification of AON levels in tissue of mdx mice.* For measuring the concentration of AONs in tissue samples, a hybridization-ligation assay based on one previously published was used.^24^ Tissues were homogenized in 100 mmol/l Tris-HCl pH 8.5, 200 mmol/l NaCl, 0.2% sodium dodecyl sulfate, 5 mmol/l ethylenediaminetetraacetic acid, and 2 mg/ml proteinase K using zirconium beads (1.4 mm; OPS Diagnostics, Lebanon, NJ) in a MagNA Lyser (Roche Diagnostics). Samples were diluted 600 and 6,000 times (muscle) or 6,000 and 60,000 (liver and kidney) in pooled control *mdx* tissue in PBS. Calibration curves of the analyzed exon 23AONs prepared in 60 times pooled control *mdx* tissue in PBS were included. All analyses were performed in duplicate.

*Statistical analyses.* A Student's *T*-test was used to determine significant differences in exon skipping levels, AON levels, plasma protein levels, and spleen/bodyweight ratios. Mixed model linear regression analysis was used to determine significant differences in bodyweight over time. Results were deemed significantly different when *P* <0.05.

**SUPPLEMENTARY MATERIAL** **Table S1.** Overview of antisense oligonucleotides. **Figure S1.** Chemical structure of 2'-O-methyl-phosphorothioate (left) and 2'-fluoro-phosphorothioate nucleobases (right). **Figure S2.** Dystrophin analysis by western blot of 23F and 23M-treated *mdx* mice.

## Supplementary Material

Supplementary Figure S1 Chemical structure of 2'-O-methyl-phosphorothioate (left) and 2'-fluoro-phosphorothioate nucleobases (right).

Supplementary Figure S2 Dystrophin analysis by western blot of 23F and 23M-treated *mdx* mice.

Supplementary Table S1 Overview of antisense oligonucleotides.

## References

[bib1] Moat, SJ, Bradley, DM, Salmon, R, Clarke, A and Hartley, L (2013). Newborn bloodspot screening for Duchenne muscular dystrophy: 21 years experience in Wales (UK). Eur J Hum Genet 21: 1049–1053. 23340516 10.1038/ejhg.2012.301 PMC3778339

[bib2] Emery, AE (2002). The muscular dystrophies. Lancet 359: 687–695. 11879882 10.1016/S0140-6736(02)07815-7

[bib3] Monaco, AP, Bertelson, CJ, Liechti-Gallati, S, Moser, H and Kunkel, LM (1988). An explanation for the phenotypic differences between patients bearing partial deletions of the DMD locus. Genomics 2: 90–95. 3384440 10.1016/0888-7543(88)90113-9

[bib4] Muntoni, F, Torelli, S and Ferlini, A (2003). Dystrophin and mutations: one gene, several proteins, multiple phenotypes. Lancet Neurol 2: 731–740. 14636778 10.1016/s1474-4422(03)00585-4

[bib5] Aartsma-Rus, A, Janson, AA, Kaman, WE, Bremmer-Bout, M, den Dunnen, JT, Baas, F et al. (2003). Therapeutic antisense-induced exon skipping in cultured muscle cells from six different DMD patients. Hum Mol Genet 12: 907–914. 12668614 10.1093/hmg/ddg100

[bib6] Aartsma-Rus, A (2010). Antisense-mediated modulation of splicing: therapeutic implications for Duchenne muscular dystrophy. RNA Biol 7: 453–461. 20523110 10.4161/rna.7.4.12264

[bib7] Järver, P, O'Donovan, L and Gait, MJ (2014). A chemical view of oligonucleotides for exon skipping and related drug applications. Nucleic Acid Ther 24: 37–47. 24171481 10.1089/nat.2013.0454 PMC3923385

[bib8] Cirak, S, Arechavala-Gomeza, V, Guglieri, M, Feng, L, Torelli, S, Anthony, K et al. (2011). Exon skipping and dystrophin restoration in patients with Duchenne muscular dystrophy after systemic phosphorodiamidate morpholino oligomer treatment: an open-label, phase 2, dose-escalation study. Lancet 378: 595–605. 21784508 10.1016/S0140-6736(11)60756-3 PMC3156980

[bib9] Mendell, JR, Rodino-Klapac, LR, Sahenk, Z, Roush, K, Bird, L, Lowes, LP et al.; Eteplirsen Study Group. (2013). Eteplirsen for the treatment of Duchenne muscular dystrophy. Ann Neurol 74: 637–647. 23907995 10.1002/ana.23982

[bib10] Goemans, NM, Tulinius, M, van den Akker, JT, Burm, BE, Ekhart, PF, Heuvelmans, N et al. (2011). Systemic administration of PRO051 in Duchenne's muscular dystrophy. N Engl J Med 364: 1513–1522. 21428760 10.1056/NEJMoa1011367

[bib11] Aartsma-Rus, A (2014) Dystrophin analysis in clinical trials. JND; 1: 41–53. 27858668

[bib12] Flanigan, KM, Voit, T, Rosales, XQ, Servais, L, Kraus, JE, Wardell, C et al. (2014). Pharmacokinetics and safety of single doses of drisapersen in non-ambulant subjects with Duchenne muscular dystrophy: results of a double-blind randomized clinical trial. Neuromuscul Disord 24: 16–24. 24321374 10.1016/j.nmd.2013.09.004 PMC4145871

[bib13] Rigo, F, Hua, Y, Chun, SJ, Prakash, TP, Krainer, AR and Bennett, CF (2012). Synthetic oligonucleotides recruit ILF2/3 to RNA transcripts to modulate splicing. Nat Chem Biol 8: 555–561. 22504300 10.1038/nchembio.939 PMC5021312

[bib14] Aartsma-Rus, A, Kaman, WE, Weij, R, den Dunnen, JT, van Ommen, GJ and van Deutekom, JC (2006). Exploring the frontiers of therapeutic exon skipping for Duchenne muscular dystrophy by double targeting within one or multiple exons. Mol Ther 14: 401–407. 16753346 10.1016/j.ymthe.2006.02.022

[bib15] Mann, CJ, Honeyman, K, McClorey, G, Fletcher, S and Wilton, SD (2002). Improved antisense oligonucleotide induced exon skipping in the mdx mouse model of muscular dystrophy. J Gene Med 4: 644–654. 12439856 10.1002/jgm.295

[bib16] Sicinski, P, Geng, Y, Ryder-Cook, AS, Barnard, EA, Darlison, MG and Barnard, PJ (1989). The molecular basis of muscular dystrophy in the mdx mouse: a point mutation. Science 244: 1578–1580. 2662404 10.1126/science.2662404

[bib17] ‘t Hoen, PA, de Meijer, EJ, Boer, JM, Vossen, RH, Turk, R, Maatman, RG et al. (2008) Generation and characterization of transgenic mice with the full-length human DMD gene. J Biol Chem; 283: 5899–5907. 18083704 10.1074/jbc.M709410200

[bib18] Heemskerk, H, de Winter, C, van Kuik, P, Heuvelmans, N, Sabatelli, P, Rimessi, P et al. (2010). Preclinical PK and PD studies on 2'-O-methyl-phosphorothioate RNA antisense oligonucleotides in the mdx mouse model. Mol Ther 18: 1210–1217. 20407428 10.1038/mt.2010.72 PMC2889733

[bib19] Shen, W, Liang, XH, Sun, H and Crooke, ST (2015). 2'-Fluoro-modified phosphorothioate oligonucleotide can cause rapid degradation of P54nrb and PSF. Nucleic Acids Res 43: 4569–4578. 25855809 10.1093/nar/gkv298 PMC4482069

[bib20] Kawasaki, AM, Casper, MD, Freier, SM, Lesnik, EA, Zounes, MC, Cummins, LL et al. (1993). Uniformly modified 2'-deoxy-2'-fluoro phosphorothioate oligonucleotides as nuclease-resistant antisense compounds with high affinity and specificity for RNA targets. J Med Chem 36: 831–841. 8464037 10.1021/jm00059a007

[bib21] Davis, S, Propp, S, Freier, SM, Jones, LE, Serra, MJ, Kinberger, G et al. (2009). Potent inhibition of microRNA *in vivo* without degradation. Nucleic Acids Res 37: 70–77. 19015151 10.1093/nar/gkn904 PMC2615630

[bib22] Zhu, CH, Mouly, V, Cooper, RN, Mamchaoui, K, Bigot, A, Shay, JW et al. (2007). Cellular senescence in human myoblasts is overcome by human telomerase reverse transcriptase and cyclin-dependent kinase 4: consequences in aging muscle and therapeutic strategies for muscular dystrophies. Aging Cell 6: 515–523. 17559502 10.1111/j.1474-9726.2007.00306.x

[bib23] Rosenblatt, JD, Lunt, AI, Parry, DJ and Partridge, TA (1995). Culturing satellite cells from living single muscle fiber explants. In Vitro Cell Dev Biol Anim 31: 773–779. 8564066 10.1007/BF02634119

[bib24] Yu, RZ, Baker, B, Chappell, A, Geary, RS, Cheung, E and Levin, AA (2002). Development of an ultrasensitive noncompetitive hybridization-ligation enzyme-linked immunosorbent assay for the determination of phosphorothioate oligodeoxynucleotide in plasma. Anal Biochem 304: 19–25. 11969184 10.1006/abio.2002.5576

