# Supplementary figures and images for: Evaluation of 2'-Deoxy-2'-fluoro Antisense Oligonucleotides for Exon Skipping in Duchenne Muscular Dystrophy
Source: Mol Ther Nucleic Acids. 2015 Dec 1;4(12):e265–. doi: 10.1038/mtna.2015.39 (PMC5014533; doi:10.1038/mtna.2015.39)

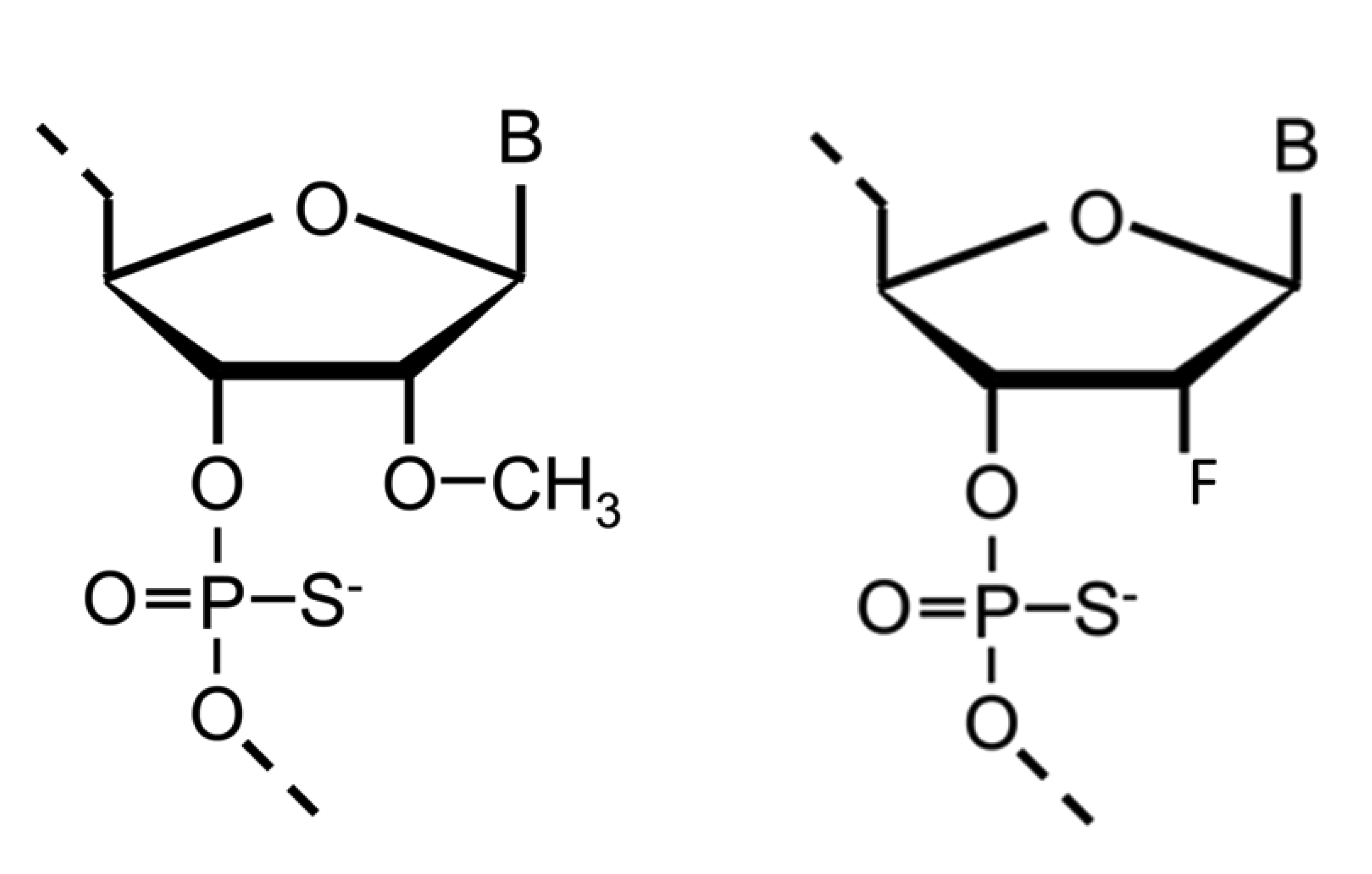

Supplement: Supplementary Figure S1 — Chemical structure of 2'-O-methyl-phosphorothioate (left) and 2'-fluoro-phosphorothioate nucleobases (right). [file mtna201539x1.tiff]

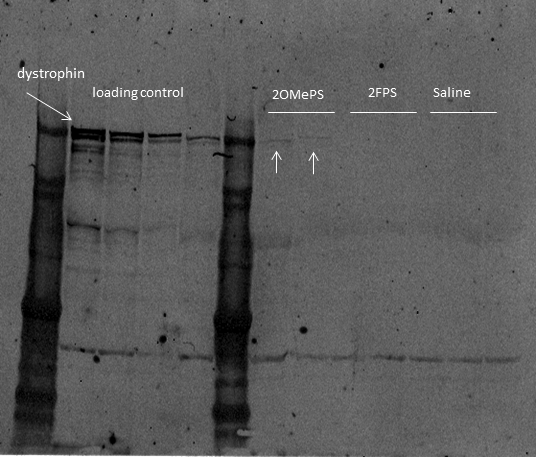

Supplement: Supplementary Figure S2 — Dystrophin analysis by western blot of 23F and 23M-treated mdx mice. [file mtna201539x2.tiff]
