# Supplementary material for: Evaluation of 2'-Deoxy-2'-fluoro Antisense Oligonucleotides for Exon Skipping in Duchenne Muscular Dystrophy
Source: Mol Ther Nucleic Acids. 2015 Dec 1;4(12):e265–. doi: 10.1038/mtna.2015.39 (PMC5014533; doi:10.1038/mtna.2015.39)
Supplement: Supplementary Table S1 — Overview of antisense oligonucleotides. [file mtna201539x3.docx]

**Suppl. Table 1**

| **AON** | **sequence** | **target** | **chemistry** |
| --- | --- | --- | --- |
| 45-1M | GCC CAA UGC CAU CCU GG | Human exon 45 | 2OMePS |
| 45-2M | UUG CCG CUG CCC AAU GCC AUC CUG G | Human exon 45 | 2OMePS |
| 45-3M | UUU GCA GAC CUC CUG CC | Human exon 45 | 2OMePS |
| 45-4M | GUU GCA UUC AAU GUU CUG AC | Human exon 45 | 2OMePS |
| 45-1F | GCC CAA UGC CAU CCU GG | Human exon 45 | 2FPS |
| 45-2F | UUG CCG CUG CCC AAU GCC AUC CUG G | Human exon 45 | 2FPS |
| 45-3F | UUU GCA GAC CUC CUG CC | Human exon 45 | 2FPS |
| 45-4F | GUU GCA UUC AAU GUU CUG AC | Human exon 45 | 2FPS |
| 53-1M | AUU GUG UUG AAU CCU UUA AC | Human exon 53 | 2OMePS |
| 53-2M | CUG UUG CCU CCG GUU CUG | Human exon 53 | 2OMePS |
| 53-3M | CCU AAG ACC UGC UCA GCU UCU UCC U | Human exon 53 | 2OMePS |
| 53-4M | AGC UUC CAG CCA UUG UGU UGA AUC C | Human exon 53 | 2OMePS |
| 53-1F | AUU GUG UUG AAU CCU UUA AC | Human exon 53 | 2FPS |
| 53-2F | CUG UUG CCU CCG GUU CUG | Human exon 53 | 2FPS |
| 53-3F | CCU AAG ACC UGC UCA GCU UCU UCC U | Human exon 53 | 2FPS |
| 53-4F | AGC UUC CAG CCA UUG UGU UGA AUC C | Human exon 53 | 2FPS |
| 23M | CGC CAA ACC UCG GCU UAC CU | Mouse exon 23 | 2OMePS |
| 23F | CGC CAA ACC UCG GCU UAC CU | Mouse exon 23 | 2FPS |
